# The predictive value of surrogate insulin resistance indices for T2DM complicated with metabolic syndrome: a retrospective study based on hospitalized patients in China

**DOI:** 10.3389/fendo.2026.1782071

**Published:** 2026-03-02

**Authors:** Sixu Xin, Xiaomei Zhang, Xin Zhao, Jianbin Sun

**Affiliations:** Department of Endocrinology, Peking University International Hospital, Beijing, China

**Keywords:** insulin resistance, metabolic syndrome, prediction model, triglyceride glucose-body mass index, triglyceride to high-density lipoprotein cholesterol ratio, triglyceride-glucose index, type 2 diabetes mellitus

## Abstract

**Objectives:**

To evaluate the predictive value of surrogate indices of insulin resistance (IR)- specifically, the triglyceride-glucose (TyG) index, the triglyceride glucose-body mass (TyG-BMI) index, and the triglyceride (TG) to high-density lipoprotein cholesterol (HDL-C) for metabolic syndrome (MetS) in patients with type 2 diabetes mellitus (T2DM).

**Methods:**

A single-center, retrospective study was conducted involving 2409 T2DM patients. Based on the presence of MetS, participants were divided into a T2DM-MetS group (n=1,787) and a T2DM-only group (n=622). Logistic regression was used to analyze the influencing factors for T2DM complicated with MetS, and to compare the predictive value of the TyG index, the TyG-BMI index, and the TG/HDL-C ratio. A nomogram prediction model was constructed. The model’s discriminative ability, clinical utility, and calibration were evaluated using the receiver operating characteristic (ROC) curve, decision curve analysis (DCA), and a calibration curve, respectively.

**Results:**

The multivariate logistic regression analysis model revealed that Sex, Wasit-to-hip ratio (WHR), fasting C-Peptide (FCP), 2-hour C-Peptide (2hCP), the TyG index, the TyG-BMI index, and the TG/HDL-C ratio were risk factors for T2DM complicated with MetS. The area under the curve (AUC) for the TyG index, the TyG-BMI index, and the TG/HDL-C ratio in predicting T2DM complicated with MetS were 0.809, 0.807, and 0.915, respectively. The prediction model was constructed using the TG/HDL-C ratio, Sex, WHR, and FCP. The model demonstrated that the C-index for predicting the presence of MetS in T2DM patients was 0.922 (95% CI: 0.909, 0.936). The DCA showed a maximum net benefit rate of 0.742.

**Conclusions:**

The surrogate indices for IR (the TyG index, the TyG-BMI index, and the TG/HDL-C ratio) were risk factors for T2DM complicated with MetS, among which the TG/HDL-C ratio was the optimal predictor. The nomogram model constructed based on the TG/HDL-C ratio, Sex, WHR, and FCP demonstrated good predictive performance for T2DM complicated with MetS. This model shows good calibration and practicality, providing a valuable reference to aid in early identification and preventive strategies in clinical practice.

## 1 Introduction

MetS is defined as a multifactorial and multicomponent clinical syndrome characterized by metabolically interconnected disorders, with core features including obesity, hyperglycemia, dyslipidemia (such as hypertriglyceridemia and low HDL-C), and hypertension (HTN). MetS not only directly promotes the development of atherosclerotic cardiovascular disease (ASCVD) but also increases the risk of T2DM. Consequently, individuals with MetS are considered a high-risk population for cardiovascular disease (CVD) (1). Given that IR is central to its pathogenesis, MetS shares key features with T2DM, including IR itself, HTN, and glucolipid metabolic disorders. Furthermore, the prevalence of both conditions has been rising steadily year by year (2). This contributes to a higher risk of CVD and patient mortality, thereby creating significant pressure on public health systems (3). In recent years, researchers have consequently dedicated efforts to discovering biomarkers for diagnosing and monitoring T2DM complicated with MetS. Such biomarkers hold promise for the early identification of high-risk individuals, assessment of disease progression, and formulation of personalized treatment strategies, which could facilitate early intervention, delay dis-ease progression, lower complication rates, and ultimately enhance patient quality of life and prognosis.

HOMA-IR serves as a simple surrogate marker for IR. However, due to limitations in health care resources and technical capabilities in some regions of China, the measurement of insulin has not yet been widely implemented. Given these limitations, using IR-sensitive biomarkers as clinical tools for evaluating metabolic health may offer practical advantages in research and clinical practice. Currently utilized to evaluate IR, the TyG index is a crucial indicator of HOMA-IR derived from TG and fasting plasma glucose (FPG) levels, which as a non-insulin-based marker, presents advantages over HOMA-IR in terms of lower cost and easier clinical implementation (4). Recent studies suggest that the TyG-BMI index may serve as a potential alternative measure of IR, which combines the TyG index and BMI. This composite indicator incorporates the influence of obesity on IR, thereby providing a more comprehensive assessment of IR severity (5). Moreover, the TG/HL-C ratio has been previously validated as a surrogate measure of IR (6).

Therefore, this study aims to evaluate the predictive value of the TyG index, the TyG-BMI index, and the TG/HDL-C ratio for T2DM complicated with MetS, with the goal of identifying the optimal parameter to serve as a practical tool for clinical diagnosis and management, thereby offering a simpler, more cost-effective, and efficient method.

## 2 Materials and methods

### 2.1 Ethics statement

The study was approved by the Ethics Committee of the Peking University International Hospital and was conducted in accordance with the ethics standards of institutional and national research committees and the 1964 Helsinki Declaration and its later amendments or comparable ethics standards. The study was a retrospective analysis; therefore, the requirement for written informed consent was waived.

### 2.2 Research subjects

This single-center, retrospective study enrolled patients diagnosed with T2DM who were hospitalized in Peking University International Hospital between March 2015 and August 2021.

The inclusion criteria were as follows: (1) aged 18 years or older, and (2) meeting the diagnostic criteria for T2DM (7); (3) meeting the diagnostic criteria for MetS.

Exclusion criteria included: (1) type 1 diabetes mellitus or other diabetic types; (2) severe hepatic or renal dysfunction; (3) acute diabetic complications or active acute/chronic infections; (4) the presence of other endocrine diseases, such as Cushing’s syndrome, thyroid dysfunction (hyper- or hypothyroidism), hyperparathyroidism; (5) pregnancy or lactation.

### 2.3 Methods

#### 2.3.1 Clinical conditions

The following data were collected for all study participants: age, duration of DM, height, weight, systolic blood pressure (SBP), diastolic blood pressure (DBP), waist circumference (WC), and hip circumference (HC). BMI= Weight (kg)/Height (m²). WHR =WC (cm)/HC (cm).

#### 2.3.2 Laboratory biochemical indices

All laboratory biochemical parameters were measured by the Clinical Laboratory of Peking University International Hospital. Blood samples for fasting parameters (including FPG, fasting insulin (FINS), FCP, lipid profile, liver function, and renal function) and glycated hemoglobin (HbA1c) were collected between 6:00 AM and 7:00 AM following an overnight fast. All participants were hospitalized and received standardized meals provided by the hospital’s nutritional kitchen. Dinner was served at approximately 5:30 PM, and no additional food intake was permitted thereafter until blood collection the next morning unless medically required. This ensured a consistent fasting duration of approximately 12–13 hours across the entire cohort. Samples for 2hPPG, 2hINS, and 2hCP were collected 120 minutes after a standardized meal. The TyG index was calculated using the formula: ln [TG (mg/dL) × FPG (mg/dL)/2]. The TyG-BMI index was calculated using the formula: TyG × BMI (kg/m²).

#### 2.3.3 MS diagnostic criteria

MetS was diagnosed according to the Guideline for the prevention and treatment of diabetes mellitus in China (2024 edition) (8) and the revised definition of abdominal obesity for the Chinese population (9). The presence of at least three of the following criteria was required for a diagnosis of MetS:

1. Abdominal obesity (i.e., central obesity): waist circumference ≥90 cm in men or ≥85 cm in women;
2. Hyperglycemia: FPG ≥6.1 mmol/L, or 2-hour plasma glucose during an oral glucose tolerance test (OGTT) ≥7.8 mmol/L, and/or previously diagnosed diabetes under treatment;
3. Elevated blood pressure: blood pressure ≥130/85 mmHg (1 mmHg = 0.133 kPa) and/or previously diagnosed hypertension under treatment;
4. Fasting triglycerides (TG) ≥1.70 mmol/L;
5. Fasting HDL-C <1.04 mmol/L.

### 2.4 Statistical analysis

All statistical analyses were performed using SPSS version 31.0 software and R 4.2.1 software. Continuous variables with a normal distribution and homogeneity of variance are presented as the mean ± standard deviation (x¯ ± s), and comparisons among multiple groups were conducted using analysis of variance (ANOVA). Data with a skewed distribution were expressed as median (interquartile range). Differences between groups were compared using the Mann-Whitney U test. Qualitative data are expressed as percentages (%). The chi-square test was used to compare the qualitative data among the groups. Univariable and multivariable logistic regression analyses were performed to identify factors associated with MetS in T2DM patients. The predictive value of the TyG index, TyG-BMI index, and the TG/HDL-C ratio for T2DM with MetS was evaluated using ROC curves and the AUC, with pairwise comparisons between indices. The optimal predictive indicator, combined with other key variables, was used to construct a predictive model. The probability (P) of MetS in T2DM occurrence was calculated using the formula: P = 1/[1 + exp(-LP)], where LP denotes the linear predictor. The predictive accuracy of the model was assessed by the concordance index (C-index). Internal validation of the model was performed using the bootstrap method with 1,000 resampling iterations. Finally, the clinical utility and calibration of the model were evaluated using DCA and a calibration curve, respectively. Statistical significance was set at a two-sided P-value < 0.05.

## 3 Results

### 3.1 Comparison of clinical characteristics between the two groups

Herein, 2409 T2DM patients aged ≥ 18 years were included in the study. Based on the presence of MetS, participants were divided into a T2DM-MetS group (n=1,787) and a T2DM-only group (n=622). The T2DM-MetS group comprised 64.30% males and 35.70% females. Patients complicated with MetS were younger, had a shorter disease duration, and exhibited lower HDL-C levels. Compared to those without MetS, the T2DM-MetS group showed significantly higher values in SBP, DBP, WL, WHR, BMI, ALT, AST, sCr, SUA, FPG, 2hPPG, FCP, 2hCP, TG, the TyG index, the TYG-BMI index, and the TG/HDL-C ratio (P < 0.05). For the remaining variables, no statistically significant differences were observed between the two groups (P > 0.05) (**Table 1**).

**Table 1:** Comparison of general conditions and biochemical indexes between the two groups.

Variable	T2DM-only (n=622)	T2DM-MetS (n=1787)	F (X2)	p	Variable	T2DM-only (n=622)	T2DM-MetS (n=1787)	F (X2)	p
Sex (%)			27.46	0.000	SUA (mmol/L)	308.24 ± 83.75	360.03 ± 92.11	152.70	0.000
Male	326 (13.53)	1149 (47.70)			FPG (mmol/L)	8.50 ± 3.46	9.14 ± 3.39	15.97	0.000
female	296 (12.29)	638 (26.48)			2hPPG (mmol/L)	12.14 ± 4.21	12.77 ± 4.28	10.28	0.001
Age (year)	58.61 ± 13.47	53.78 ± 13.84	56.88	0.000	HbA1C (%)	8.59 ± 2.09	8.73 ± 1.77	2.51	0.113
Duration of DM (year)	10.27 ± 8.47	8.58 ± 7.60	21.29	0.000	FCP (ng/ml)	1.89 ± 0.99	2.62 ± 1.29	164.87	0.000
SBP (mmHg)	128.55 ± 16.71	133.54 ± 17.64	37.98	0.000	2hCP (ng/ml)	4.83 ± 2.79	5.91 ± 3.57	47.38	0.000
DBP (mmHg)	75.27 ± 9.00	80.25 ± 11.55	95.46	0.000	FINS (uU/ml)	16.27 ± 58.16	15.69 ± 28.41	0.11	0.745
WL (cm)	89.47 ± 10.18	97.20 ± 9.23	306.24	0.000	2hINS (uU/ml)	45.25 ± 74.57	50.48 ± 53.49	3.54	0.060
WHR	0.92 ± 0.06	97.20 ± 9.23	142.81	0.000	TC (mmol/L)	4.34 ± 0.98	4.41 ± 1.14	1.98	0.160
BMI (kg/m2)	24.01 ± 3.35	26.67 ± 3.64	255.57	0.000	TG (mmol/L)	1.15 ± 0.72	2.47 ± 1.89	290.13	0.000
ALT (U/L)	21.63 ± 18.57	29.50 ± 36.38	26.71	0.000	HDL-C (mmol/L)	1.24 ± 0.24	0.92 ± 0.18	1245.72	0.000
AST (U/L)	21.11 ± 12.06	24.20 ± 18.13	15.67	0.000	LDL-C (mmol/L)	2.54 ± 0.86	2.74 ± 5.89	0.69	0.406
sCr (umol/L)	66.23 ± 25.27	71.02 ± 30.27	12.54	0.000	TyG	8.80 ± 0.58	9.57 ± 0.70	602.42	0.000
eGFR	96.46 ± 18.50	97.76 ± 20.52	1.96	0.162	TyG-BMI	211.48 ± 33.70	255.35 ± 41.12	573.82	0.000
UACR (mg/g)	71.81 ± 374.34	94.31 ± 361.41	1.756	0.185	TG/HDL	0.98 ± 0.68	2.89 ± 2.80	284.62	0.000
					HOMA-IR	6.17 ± 24.30	6.30 ± 9.79	0.031	0.860

### 3.2 Logistic regression analysis in patients with T2DM complicated with MetS

Multivariate logistic regression analysis, incorporating significant univariate predictors (from **Table 1**) and complicated with MetS (yes=1/no=0) as the dependent variable, revealed that the TyG index, the TyG-BMI index, the TG/HDL-C ratio, Sex, WHR, FCP, and 2hCP were independently associated with MetS in T2DM patients (P < 0.05). (**Table 2**) T2DM patients were divided into five groups based on the number of MetS components at baseline: Q1 (T2DM alone), Q2 (T2DM + 1 MetS component), Q3 (T2DM + 2 MetS components), Q4 (T2DM + 3 MetS components), and Q5 (T2DM + 4 MetS components). The results showed that as the clustering of MetS components increased, the levels of the TyG index, the TyG-BMI index, and the TG/HDL-C ratio also increased. Pairwise comparisons revealed that the TyG index and the TyG-BMI index differed significantly among all five groups (P < 0.05). All pairwise comparisons of the TG/HDL-C ratio were statistically significant (P < 0.05), except for the comparison between Group 1 and Group 2 (P > 0.05) (**Table 3**).

**Table 2:** Logistic regression analysis of patients with T2DM complicated with MetS.

Variables	Univariate logistics regression		Multivariate logistics regression	
	OR (95%CI)	P	OR (95%CI0.13)	P for interaction
TyG	7.029 (5.787, 8.538)	0.000	6.049 (4.8877, 7.489)	0.000
TyG-BMI	1.036 (1.033, 1.040)	0.000	1.030 (1.026, 1.034)	0.000
TG/HDL	17.036 (13.133, 22.100)	0.000	14.795 (11.283, 19.401)	0.000
Sex	0.612 (0.508, 0.736)	0.000	0.724 (0.546, 0.960)	0.025
Age	0.974 (0.968, 0.981)	0.000	1.004 (0.992, 1.015)	0.545
Duration of DM	0.974 (0.963, 0.985)	0.000	1.002 (0.984, 1.021)	0.809
SUA	1.007 (1.006, 1.008)	0.000	1.001 (0.999, 1.003)	0.231
WHR	16168,896 (2988.179, 87489,121)	0.000	103.794 (8.096, 1330,774)	0.000
FCP	1.933 (1.740, 2.147)	0.000	1.212 (1.027, 1.431)	0.023
2hCP	1.123 (1.086, 1.161)	0.000	0.947 (0.898, 0.998)	0.042
ALT	1.024 (1.016, 1.031)	0.000	1.002 (0.992, 1.012)	0.679
AST	1.017 (1.009, 1.026)	0.000	1.001 (0.987,1.016)	0.862
sCr	1.009 (1.004, 1.013)	0.000	0.999 (0.994,1.005)	0.854

**Table 3:** Comparison of numbers of MetS components and IR replacement index.

Variable	Numbers of MetS components*						
	Q_1_	Q_2_	Q_3_	Q_4_	Q_5_	F	P
TyG^a^	8.65 ± 0.56	8.84 ± 0.57	9.24 ± 0.65	9.75 ± 0.64	10.00 ± 0.63	275.583	0.000
TyG-BMI^b^	185.85 ± 26.14	218.92 ± 31.97	237.56 ± 33.64	263.29 ± 38.41	287.07 ± 45.98	293.751	0.000
TG/HDL-C^c^	0.78 ± 0.32*	1.03 ± 0.74	1.88 ± 1.43	3.43 ± 3.07	4.38 ± 3.91	149.485	0.000

*T2DM patients were divided into five groups based on the number of MetS components at baseline: Q1 (T2DM alone), Q2 (T2DM + 1 MetS component), Q3 (T2DM + 2 MetS components), Q4 (T2DM + 3 MetS components), and Q5 (T2DM + 4 MetS components). ^a^All pairwise comparisons of the TyG index were statistically significant (P < 0.05). ^b^All pairwise comparisons of the TyG-BMI index were statistically significant (P < 0.05). ^c^All pairwise comparisons of the TG/HDL-C ratio were statistically significant (P < 0.05), except for the comparison between Group 1 and Group 2 (P > 0.05).

### 3.3 The predictive value of different IR surrogate indices for MetS in T2DM patients

**Figure 1:**
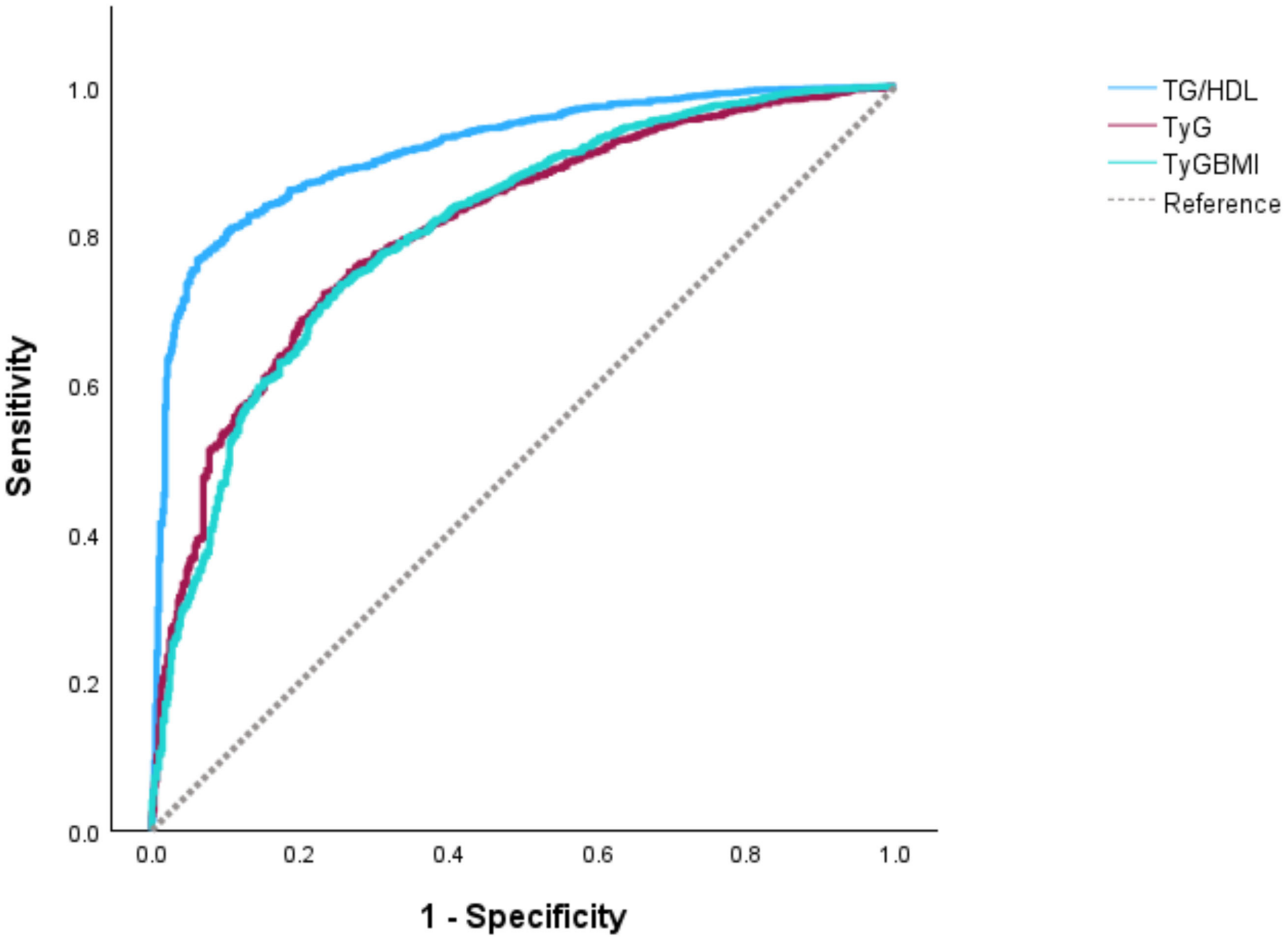
ROC curves of the TyG index, the TyG-BMI index and the TG/HDL-C ratio predicting MetS in T2DM patients. The AUC for the surrogate indices of insulin resistance in predicting MetS were 0.809, 0.807, and 0.915, sequentially, among which the TG/HDL-C ratio was the optimal predictor.

To assess the diagnostic performance, ROC curves were constructed using the TyG index, the TyG-BMI index, and the TG/HDL-C ratio as test variables, with MetS status (presence=1, absence=0) in T2DM patients as the classification variable. The AUC for the TyG index, the TyG-BMI index, and the TG/HDL-C ratio in predicting T2DM complicated with MetS were 0.809, 0.807, and 0.915, respectively, among which the TG/HDL-C ratio was the optimal predictor. DeLong’s test revealed significant differences in AUC between the TG/HDL-C ratio and both the TyG index (P < 0.001) and the TyG-BMI index (P < 0.001), while no significant difference was found between the TyG index and the TyG-BMI index (P = 0.849) (**Table 4**; **Figure 1**).

**Table 4:** The predictive value of different insulin resistance surrogate indices for MetS (AUC, Optimal Cut-off Value, Sensitivity, Specificity, and Youden index).

	AUC	95% CI	P	Optimal cut-off value	Sensitivity (%)	Specificity (%)	Youden index
TyG	0.809	0.790, 0.828	0.000	9.171	72.2	76.7	0.489
TyG-BMI	0.807	0.787, 0.826	0.000	230.780	73.4	74.6	0.479
TG/HDL	0.915	0.903, 0.927	0.000	1.562	76.7	93.9	0.706

### 3.4 Predictive performance of the TG/HDL-C ratio combined with other key indicators for T2DM complicated with MetS

**Figure 2:**
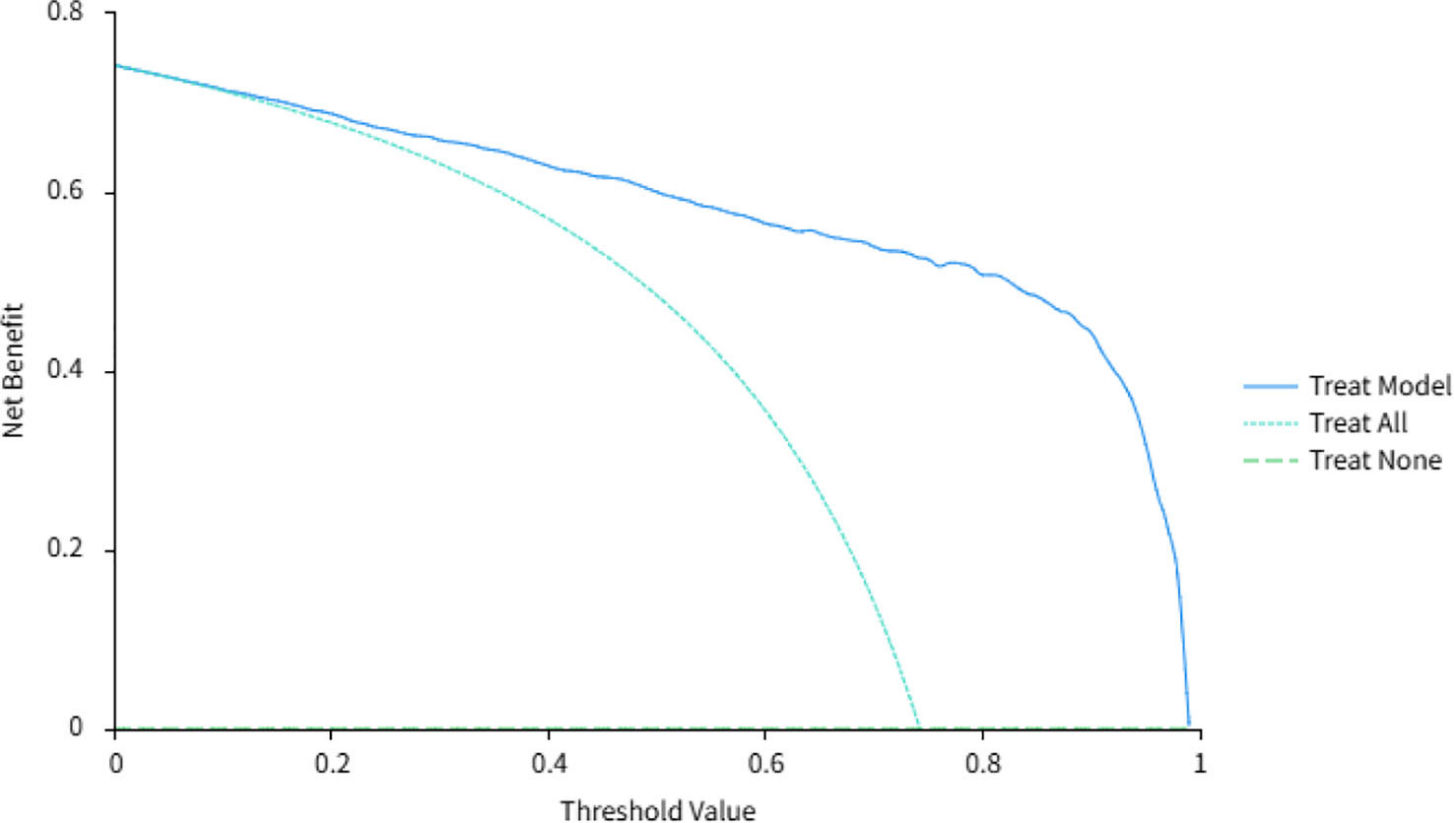
Decision curve analysis of prediction mode. The results showed that within a risk threshold range of 0.00 to 1.00, the model achieved a maximum net benefit of 0.742.

**Figure 3:**
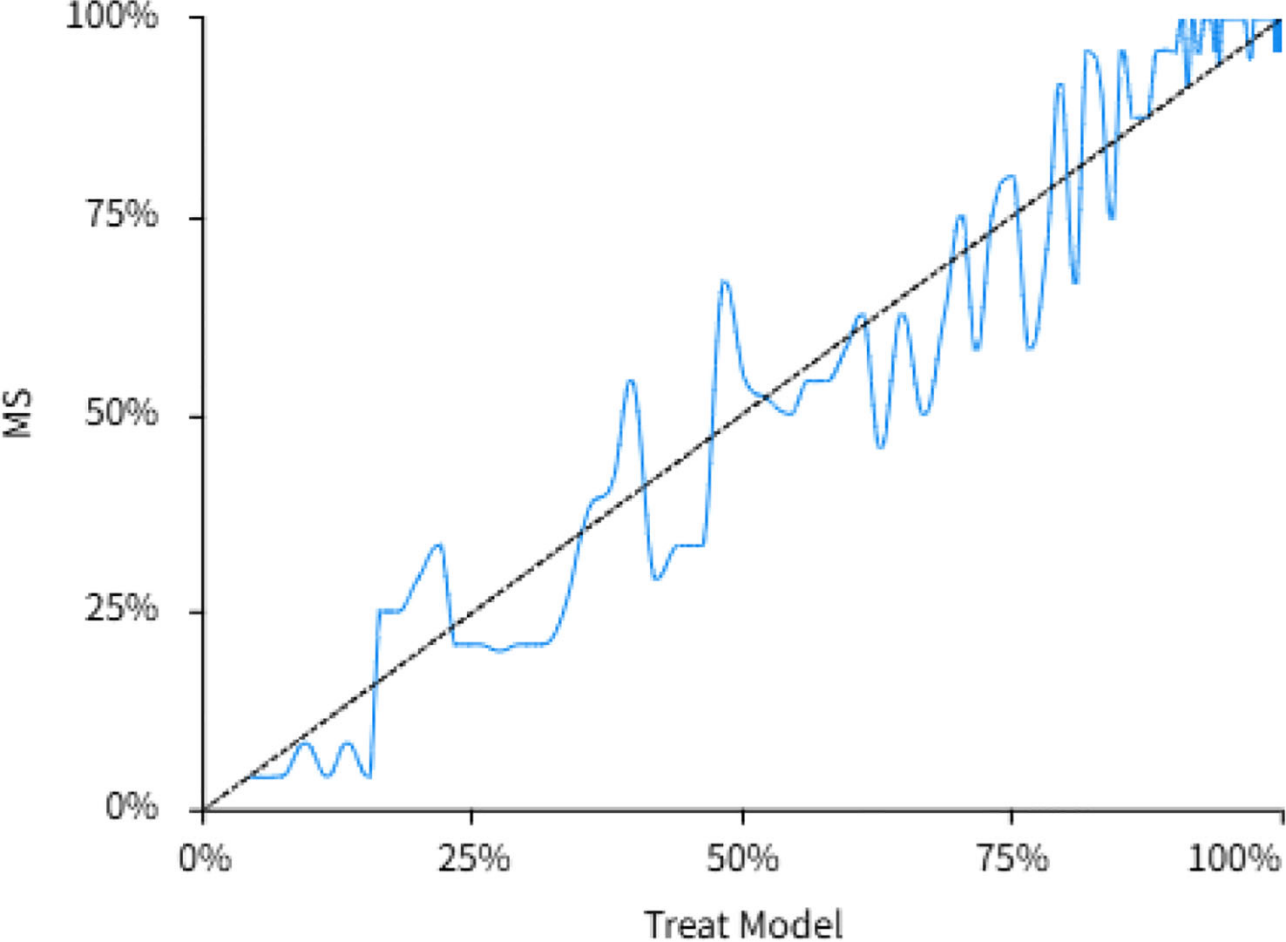
Calibration curve analysis of prediction mode. The results indicated a close alignment of predictions with actual observations.

Based on the results of multivariate logistic regression analysis, a prediction model for T2DM complicated with MetS was constructed using the TG/HDL-C ratio, Sex, WHR, and FCP. The probability (P) of developing comorbid MetS was calculated using the formula: P = 1/[1 + exp(-LP)], where the linear predictor (LP) was: LP = −10.53 − 0.278 × Sex (coded as 2) + 7.445 × WHR + 0.257 × FCP + 2.842 × TG/HDL-C. The model demonstrated that the C-index for predicting the presence of MetS in T2DM patients was 0.922 (95% CI: 0.909, 0.936). Internal validation using the bootstrap method with 1000 resamples yielded statistically significant results, indicating that the established model maintained good predictive performance in the validation sets. The DCA was performed to evaluate the clinical net benefit. The results showed that within a risk threshold range of 0.00 to 1.00, the model achieved a maximum net benefit of 0.742, supporting its favorable clinical utility. Similarly, the calibration curve indicated a close alignment of predictions with actual observations, suggesting good model calibration. (**Figures 2**, **3**).

## 4 Discussion

A cohort of 2049 adults with T2DM was included. The overall MetS prevalence was 74.18% (males: 64.30%; females: 35.70%). The T2DM-MetS group demonstrated notably higher values in SBP, DBP, WL, WHR, BMI, TG, SUA, FPG, 2hPPG, FCP, and 2hCP compared to the T2DM-only group. Additionally, the TyG index, TyG-BMI index, and the TG/HDL-C ratio were significantly elevated in the T2DM-MetS group and as the clustering of MetS components increased, the levels of the TyG index, the TyG-BMI index, and the TG/HDL-C ratio also increased. Multivariate logistic regression con-firmed that these three indices were independent factors associated with MetS in T2DM. Notably, in contrast to the surrogate indices evaluated, HOMA-IR did not differ significantly between T2DM patients with and without MetS in our cohort. This finding may reflect the confounding influence of glucose-lowering therapies, particularly insulin and insulin secretagogues, which can elevate circulating insulin levels independent of underlying insulin resistance. This further supports the utility of non-insulin-based markers such as the TG/HDL-C ratio in clinical settings where insulin measurement is impractical or influenced by pharmacotherapy.

This is the first study to comprehensively evaluate the relationship and diagnostic capabilities of three IR indices (TyG, TyG-BMI, and the TG/HDL-C ratio) in relation to MetS within a cohort of 2409 T2DM patients. We discovered that among the IR indices, the strongest association with MetS was for the TG/HDL-C ratio. Multi-variate logistic regression provided additional evidence that the TG/HDL-C ratio is an independent determinant of MetS, maintaining a significant association even after adjusting for confounding variables such as age, sex, disease duration and the uric acid. In terms of diagnostic performance, ROC curve analysis revealed that the TG/HDL-C ratio demonstrated the greatest overall ability to distinguish between groups, high-lighting its potential as a non-invasive screening tool.

Earlier research has demonstrated a significant association between a higher TG/HDL-C ratio and unfavorable metabolic traits, such as dyslipidemia, obesity, diabetes, and Metabolic Dysfunction-Associated Fatty Liver Disease (MAFLD) (10–12). Recently there has been increasing attention on the association between this ratio and MetS. A cross-sectional study, involving over 5000 Iranian participants compared various lipid ratios for detection of MetS in the Iranian general population. The researchers concluded that the TG/HDL-C was the best indicator for identifying MetS compared to the other ratios (13). Another study in Iran demonstrated that the high TG/HDL-C ratio was associated with a 2.12 times increased risk of developing MetS, using a cut-off point of 4.03 for males and 2.86 for females (14). In 2021, a cross-sectional study was conducted in the elderly Chinese population, which included a total of 1267 participants ≥ 65 years of age, to investigate a correlation between TG/HDL-C ratio and MetS. They determined that TG/HDL-C ratio values exceeding the cut-off values of 1.437 for men and 1.196 for women predicted a higher risk of developing MetS (15). In line with these, data from our study on the T2DM population showed that the TG/HDL-C ratio conveyed a significantly higher risk for the diagnosis of MetS (adjusted OR = 14.795, 95% CI: 11.283- 19.401; P = 0.000), using an optimal cut-off point of 1.562. Undoubtedly, the TG/HDL-C ratio was a very satisfactory predictor for MetS in T2DM patients. Nonetheless, taking into consideration the different cut-off values of multiple trials, based on ethnicity, genetics and lifestyle, the afore mentioned ratio cannot be considered an absolute parameter without calibration.

Studies have demonstrated that the TyG index, which integrates FPG and TG, is a convenient tool for detecting IR, one of the main components of MetS (16). A me-ta-analysis and systematic review of 13 studies (N = 49,325) on the diagnostic accuracy of the TyG index for MetS in adults showed the summary ROC analysis yielded an AUC of 0.90 (79% specificity, 82% sensitivity) in males and 0.87 (85% specificity, 81% sensitivity) in females, supporting its high diagnostic accuracy for MetS (17). A large, community-based, prospective cohort over 12 years of follow-up conducted by Da-Hye Son et al. from South Korea, evaluated the comparative predictive utility of the TyG index and HOMA-IR for the prevalence and incidence of MetS (18). It was demonstrated that the TyG index showed higher predictive power for prevalent MetS than HOMA-IR, with optimal cutoffs of 8.718 for prevalence and 8.518 for incidence. Further evidence from a large-scale population-based study conducted in Wuhu, China, involving 298,652 participants, demonstrated that the prevalence of MetS showed a corresponding increase across ascending quartiles of the TyG index which also identified the optimal TyG index cutoff for diagnosing MetS to be 8.85, with a sensitivity of 81% and a specificity of 91% (19). In line with these, data from our study on the T2DM population showed that the TyG index conveyed a significantly higher risk for the diagnosis of MetS (adjusted OR = 6.049, 95% CI: 4.8877- 7.489; P = 0.000), using an optimal cut-off point of 9.171.

Notably, the TyG-BMI index, which is modified by BMI, has shown superior performance in evaluating the severity of IR. This index, calculated as Ln [TG (mg/dl) × FPG (mg/dl)/2] × BMI, was first proposed in 2016 by Er et al. (20). It was also noted that, with a range of 16.6%, the TyG-BMI index had a strong correlation with HOMA-IR among the visceral obesity indices and TyG-related values. A Study by Lim et al. not only confirmed the above findings, but further suggested that the TyG-BMI index was a more accurate predictor of IR than the TyG index and the TyG-WC index alone (21). Gui et al. revealed that among middle-aged and elderly adults, various obesity- and lipid-related indices could predict MetS, with the TyG-BMI index being the strongest predictor in males (22). Additionally, Tamini et al. demonstrated that the TyG-BMI index served as a promising non-invasive instrument to assess MetS risk in obese pediatric and adolescent populations (23). The studies reviewed above demonstrate that the TyG-BMI index could serve as an effective, non-invasive clinical biomarker for the early recognition of MetS. In line with previous researches, our findings also established the TyG-BMI index as an independent determinant of MetS within the T2DM population (adjusted OR = 1.030, 95% CI: 1.026- 1.034; P = 0.000), using an optimal cut-off point of 230.780.

Based on the optimal predictive indicator for MetS, the TG/HDL-C ratio, combined with other key indicators (sex, WHR, and FCP), a prediction model for the development of MetS in patients with T2DM was further developed in this study. The ROC curve analysis yielded an AUC of 0.922 (95% CI: 0.909, 0.936), indicating good predictive performance. The DCA analysis showed that within a risk threshold range of 0.00 to 1.00, the model achieved a maximum net benefit of 0.742, supporting its favorable clinical utility. Similarly, the calibration curve indicated a close alignment of predictions with actual observations, suggesting good model calibration. Thus, the model has significant utility for evaluating MetS risk among individuals with T2DM, guiding both clinical risk prediction and preventive strategies.

In addition to the biochemical and anthropometric indicators evaluated in this study, it is worth emphasizing that early and accurate anthropometric assessment remains a cornerstone in identifying individuals at high risk for MetS. While traditional measures such as BMI and WHR are valuable, emerging evidence highlights the importance of body fat distribution in metabolic risk stratification. For instance, recent studies suggest that echocardiographic imaging modalities- particularly speckle tracking echocardiography- can provide non-invasive insights into cardiometabolic risk. Myocardial and atrial strain parameters have been shown to correlate with MetS probability, particularly in individuals with android (central) obesity, whereas those with gynoid fat distribution exhibit lower metabolic risk (24). Integrating such imaging-based assessments with anthropometrics and biochemical indices like the TG/HDL-C ratio could facilitate a more comprehensive, multimodality approach to early risk detection and personalized prevention strategies in T2DM patients.

## 5 Conclusion

In conclusion, the surrogate indices for IR (the TyG index, the TyG-BMI index, and the TG/HDL-C ratio) were risk factors for T2DM complicated with MetS. This is the first large-sample study (n = 2,409) to systematically compare three IR surrogate indices (TyG, TyG-BMI, TG/HDL-C) specifically in a T2DM population for predicting MetS. While previous studies have investigated these indices individually or in general populations, no study has evaluated their comparative diagnostic performance in T2DM patients using a comprehensive analytic framework including ROC, DeLong’s test, and nomogram construction. We further demonstrated that TG/HDL-C ratio outperforms TyG and TyG-BMI in this specific population, and constructed a clinically applicable nomogram integrating TG/HDL-C, sex, WHR, and FCP, achieving an AUC of 0.922. This provides a cost-effective, non-insulin-dependent tool for early MetS risk stratification in T2DM patients, particularly in resource-limited settings. An important methodological strength of this study lies in the standardized fasting protocol. Unlike outpatient or community-based retrospective studies, in which fasting duration is often documented as “at least 8 hours” with considerable inter-individual variability, all participants in our cohort were hospitalized under a controlled dietary regimen. Dinner was served at a fixed time (5:30 PM), and no caloric intake was permitted thereafter until blood collection the following morning. This ensured a consistent fasting duration of approximately 12–13 hours for all subjects, thereby minimizing confounding effects of variable fasting intervals on triglyceride and glucose measurements. This design enhances the internal validity of our findings and supports the reliability of IR surrogate indices such as the TG/HDL-C ratio. However, our study has some limitations. First, this is a single center retrospective study. Future large-scale, multi-center trials are needed to establish the causal relationship between surrogate IR indices for T2DM complicated with MetS, which would provide further validation for our conclusions. Second, all participants were recruited from Beijing, China, and the MetS diagnostic criteria used were tailored to abdominal obesity features characteristic of the Chinese population, which may limit the generalizability of our findings to other regions or ethnic groups, particularly those with different body composition patterns or diagnostic thresholds. Third, this study did not systematically adjust for the confounding effects of glucose-lowering medications (such as insulin, insulin secretagogues, metformin, etc.). These drugs may directly influence insulin levels and metabolic parameters, thereby affecting the interpretation of the study’s indicators. Future research should systematically collect and analyze detailed medication data to validate the robustness and generalizability of indicators such as the TG/HDL-C ratio in treated T2DM populations.

## Data Availability

The raw data supporting the conclusions of this article will be made available by the authors, without undue reservation.
